# Supplementary material for: Patterns of Intron Gain and Loss in Fungi
Source: PLoS Biol. 2004 Nov 30;2(12):e422. doi: 10.1371/journal.pbio.0020422 (PMC532390; doi:10.1371/journal.pbio.0020422)
Supplement: Table S1 — Also available at http://genes.mit.edu/NielsenEtAl/. (4.3 MB ZIP). [file pbio.0020422.st001.zip › NielsenEtAl/html/37.html]

AN2860.1.NCU00260.1.MG00220.1.FG06619.1


```
 CLUSTAL W (1.82) Multiple Sequence Alignments - Introns Inserted


Sequence 1: AN2860.1	364 aa
Sequence 2: MG00220.1	360 aa
Sequence 3: NCU00260.1	361 aa
Sequence 4: FG06619.1	360 aa
Alignment Length: 366 aa
Number Identitical Residues: 181 aa
Alignment Score (without introns) 10047


MG00220.1 	MPYPEKFTGFQVKSPDSWTEFHKNE~FDPKPFGDYD~VDIEIECCGVCGSDVHTVS-~-G
NCU00260.1	MSVPEKFTGFQVNGPETWTEFHKNE~FQPKPFGDYD~VDIKIECCGVCGSDVHTIS-~-G
FG06619.1 	MSVPDKFQGFQSPSAEHWTDFQKNS~FEPRPFGEYD~VDIKVECCGVCASDRHTIS-~-G
AN2860.1  	MPYPEEAEGFQVDSPETYTDFKKRF0FKLKPFGDYD1VDIKIEACGVCGSDVHTISV1IG
          	*. *::  ***  ..: :*:*:*.  *. :***:** ***::*.****.** **:*   *

MG00220.1 	GWGEQKFPLVVGH-~-E1IVGKAIKVGPKVTLIKEGQRVGVGAQSYACLDCKQCKNDNET
NCU00260.1	GWGEQKFPLAVGH-~-E1IIGTALRVGPKVTLIKPGQRVGVGAQSYSCLDCRQCKNDNET
FG06619.1 	DWGACPYPLAVGH-~-E~VVGKVLRVGDKVTLAKVGDRVGAGAQVWSCMDCRQCKNDNET
AN2860.1  	GWGSQKFPLCVGHG1PE~IIGRAVRVGPKVTLIKEGQRVGVGAQSYSCGECKQCKNENET
          	.**   :** ***. .* ::* .::** **** * *:***.*** ::* :*:****:***

MG00220.1 	YC-KKQLDTYGSVWPDSGVVSQGGYSSHVRTHEHW2VFPIPDALASTIAAPMLCAGLTAY
NCU00260.1	YC-RKQLDTYGAVWPDTGIVSQGGYSSHVRTHEHW2VFPIPDALPSTVAAPMLCAGLTAY
FG06619.1 	YC-KDQIDAYGADYLDTGFKTQGGYSSHSRVHEYW2VYPIPEALSSTQAAPMLCAGITVY
AN2860.1  	YCPVLMIDTYGAEWPDTGIVSQGGYSSHVRTHEHW~VFPIPDVLETNLVAPMLCAGLTAY
          	**.   :*:**: : *:*. :******* *.**:* *:***:.* :. .*******:*.*

MG00220.1 	SPLVRNGVGPGKKVGILGMGGIGHFGLLFAKALGAEVWAISRSRAKEADCKAMGADGFLA
NCU00260.1	SPLVRNGCGPGKKVGIVGLGGIGHLGLLFAKALGATVYVISRTHSKEADARKMGADGFLA
FG06619.1 	SPLKRLGAGPGKKVGIVGIGGLGHYGVIFAKALGAEVWAISRSRAKEEDARKMGADGFLA
AN2860.1  	SPLVRNGAGPGKKVGIVGLGGIGHFGVMFAKALGAETWAISRSRAKEADARKLGADGYIA
          	*** * * ********:*:**:** *::******* .:.***:::** *.: :****::*

MG00220.1 	TSEKGWNEPHAMTFDAILNTASSFEGWDFGAYLGLLDVHGKFVAVGLPE-GEGMKIRNQD
NCU00260.1	TADPNWNEEHIMTFDLIINTANSFEGFDLDAYLSLLDVHGKWVSVGLPEDEEGIRVRNQT
FG06619.1 	TGEKNWTEGHKMTFDIIINTATSFDGFALSEYLSLLDIHGHWNSVGLPG-GDGIEIRNQD
AN2860.1  	TAEEGWEKPHRCSFDLIINCANSSKGFDLEKYLSMMDVHGRWISVGLPE-EEGQVIKAQN
          	*.: .* : *  :** *:* *.* .*: :  **.::*:**:: :****   :*  :: * 

MG00220.1 	LLSNGVFIGASHLGSRRETIEMLQLAADKGIKSWVEEIQIGEDGLKEALTRVHKTDVRYR
NCU00260.1	FLKNGCFFGSSHLGSRRETLEMLQLAADKGVRTWVEEVKINEENLKGVMQKMKGAGGRYR
FG06619.1 	FITNGCYIGTSHLGSRREMLEMLQLAADKGLKSWIEEIPISTKGLQQAMQALEKSSVRYR
AN2860.1  	LISNGVLIGASHLGSRREMLEMLQLAADKGLRGWVEELQIGSEGLKEAMERMKKGDVHYR
          	::.**  :*:******** :**********:: *:**: *. ..*: .:  :.  . :**

MG00220.1 	FCMTGYDKVFTA
NCU00260.1	FCLTGYEEAFGA
FG06619.1 	SCMTNYDEAFGA
AN2860.1  	FTMTGYDKVFG-
          	  :*.*::.*
```
